# Unveiling Druggable Pockets by Site-Specific Protein Modification: Beyond Antibody-Drug Conjugates

**DOI:** 10.3389/fchem.2020.586942

**Published:** 2020-10-21

**Authors:** Dailén G. Martínez, Stefan Hüttelmaier, Jean B. Bertoldo

**Affiliations:** Institut für Molekulare Medizin, Medizinische Fakultät, Martin-Luther-Universität Halle-Wittenberg, Halle, Germany

**Keywords:** antibody-drug conjugates, ADCs, site-selective protein modification, site-specific protein modification, drug design, targeted covalent inhibition

## Abstract

Site-specific modification approaches have been extensively employed in the development of protein-based technologies. In this field, stability and activity integrity are the envisioned features of chemically modified proteins. These methods are especially used in the design of antibody-drug conjugates (ADCs). Nevertheless, a biochemical feature of the target protein in these reactions is often overlooked, residue specificity. Usually, in the course of developing chemical probes to modify a protein of interest (POI), specific amino acids are selected due to their reactivity. It is not critical which residue is modified as long as its modification does not compromise the POI's activity. However, no attention is paid as to why certain residues are preferentially modified over others. Physicochemical and structural constraints are often involved in the reactivity of the residue and account for the preferential modification. We propose that site-specific protein modification approaches can be applied beyond the development of ADCs or protein-drug conjugates, and used as a tool to reveal functionally relevant residues. By preferentially modifying certain side chains in the POI, chemical probes can uncover new binding motifs to investigate. Here we describe methods for protein modification, and how some pitfalls in the field can be turned into tools to reveal and exploit druggable pockets. Thus, allowing the design of innovative inhibitors against disease-relevant POIs. We discuss methodologies for site-specific modification of lysine, tryptophan, cysteine, histidine and tyrosine and comment on instances where the modified residues were used as targets for functionalization or drug design.

## Introduction

**Figure 1:**
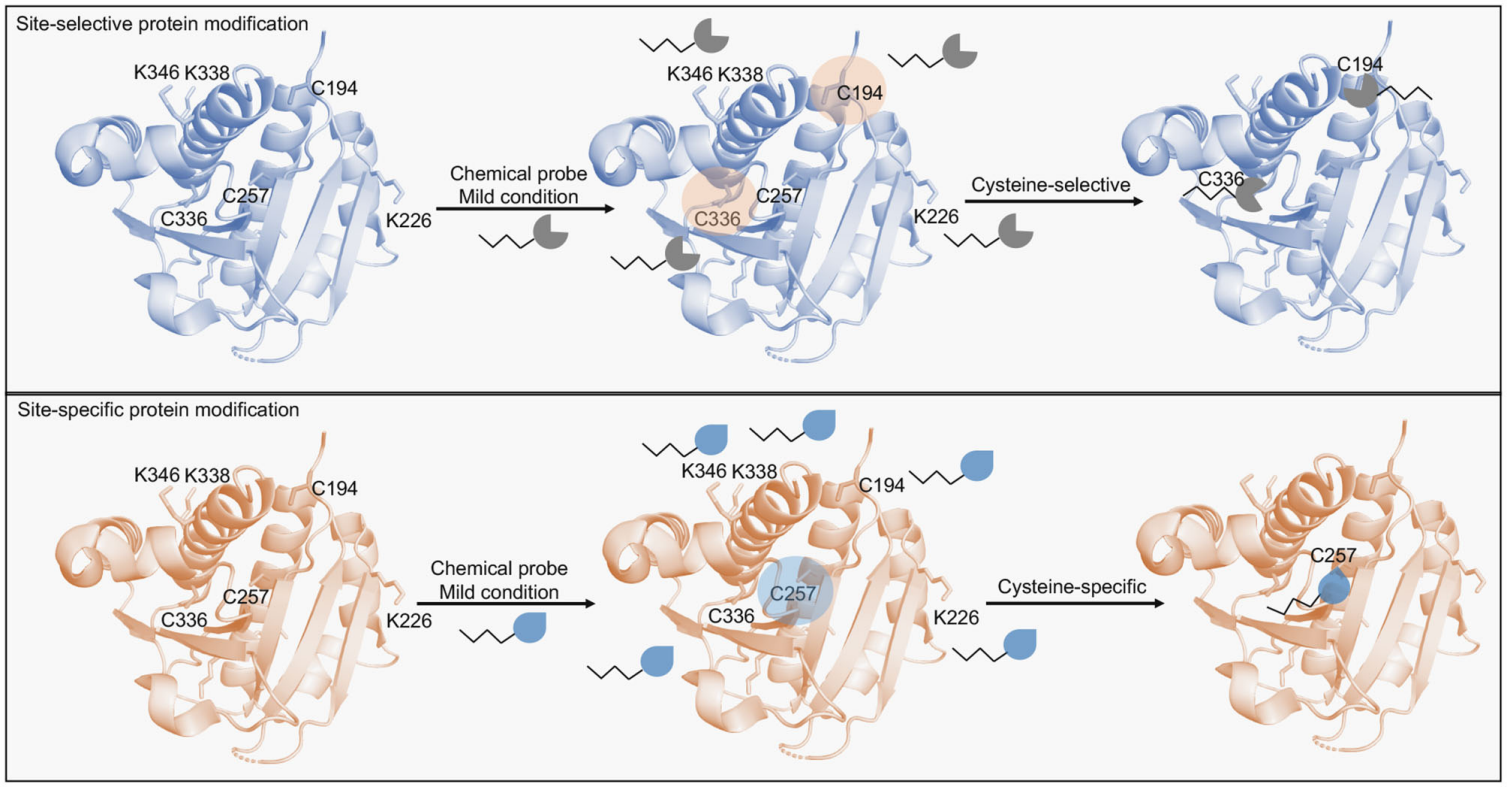
Scheme of protein modification methods targeting cysteine residues. *Site-selective protein modification* enables the selective modification of cysteine residues in a protein containing multiple highly nucleophilic residues (e.g., lysines). Multiple cysteines are usually modified by this approach. *Site-specific protein modification* in turn, enables the modification of a single residue (e.g., cysteines) in the presence of other accessible cysteine residues. Chemical probes designed for this approach are able to distinguish intrinsic cysteine reactivities based on the minute differences in the residues' pK_a_.

The chemical modification of proteins has emerged as a valuable approach to interrogate and to intervene in biological systems (Stephanopoulos and Francis, 2011). It is inspired in the natural ability of cells to induce specific post-translational modifications (PTMs) which influence the fate of protein targets and their role in cellular processes, including trafficking, signaling, migration and differentiation (Walsh et al., 2005). Consequently, understanding these processes and harnessing their potential is invaluable for therapeutic applications. Thus, it is expected that the toolbox to modify and modulate proteins would grow exponentially to encompass evermore improved and specific methodologies. These can generally be divided into site-selective modifications and site-specific modifications (Figure 1), which are often applied to study or modulate the activity of the protein target in the cellular context, also referred as biorthogonal reactions, to produce protein conjugates for cell imaging, biomaterials, or for drug delivery systems (King and Wagner, 2014; Lang and Chin, 2014; Shadish and Deforest, 2020). Except when activity modulation is the aim of a protein modification approach, methods and chemical probes are designed to allow protein functionalization without altering the structure or activity of the protein of interest (POI). These functionalization strategies are at center of protein-based technologies and have been extensively pursued in the development of protein-drug conjugates, including ADCs (Hoyt et al., 2019). However, the lack of homogenous product with a single defined modification is still a common outcome of these reactions, which can lead to loss-of-function and prevent the further development of the protein-drug conjugate. This drawback has propelled the efforts to develop several improved methods, which have been thoroughly reviewed recently (Hoyt et al., 2019; Shadish and Deforest, 2020), however, an assessment of the reasons for the loss-of-function are often not investigated or simply attributed to random modifications. In this perspective we discuss examples of site-selective and site-specific modification approaches and show the protein target as the main player rather than the chemistry used to circumvent undesired reactions. We also focus on selected cases of loss-of-function induced by site-specific chemical probes upon modification of certain residues, and propose that these probes can reveal hitherto unknown functions of their targeted residues and unveil promising “druggable pockets.” This information can then be exploited to aid drug discovery platforms aimed at the development of covalent inhibitors against disease-relevant POIs.

## Site-Selective Protein Modification

Reactions with chemical probes that can selectively modify a residue among others with similar reactivities in the POI are considered site-selective (Tadross and Jacobsen, 2012). These reactions prevent stochastic modifications by allowing the targeting of specific nucleophilic residues over others (e.g., by modifying cysteines over lysines; Spicer and Davis, 2014). These methods have substantially improved the bioconjugate chemistry field and have allowed the development of stable and active products (Kalia and Raines, 2010). Nevertheless, is relatively hard to predict which nucleophilic residue will be preferentially modified, thus, achieving a precise modification at a site that does not compromise the protein activity is essential in these reactions. Cysteine and lysines are the most common targeted residues in this approach and typically modified by maleimides and activated esters (Spicer and Davis, 2014; Koniev and Wagner, 2015; Gunnoo and Madder, 2016). Protein targets are divided based on amino acid content and distribution, with genetically inserted or naturally occurring residues. Chemical probes are then selected following the analysis of the protein target and the nature of the residue to be modified (Boutureira and Bernardes, 2015). In the genetic approach to prevent stochastic modifications, generally, a cysteine residue is inserted in a protein target, the location is critically important to avoid disrupting activity whilst also allowing access by the chemical probe. A key example is observed with RNAse A, a folic acid derivative bromoalkyl group was conjugated to an inserted cysteine residue and allowed the production of a stable conjugate able to specifically target cancer cells (Smith et al., 2011). Interestingly, RNase A has eight naturally occurring cysteine residues which form four disulfide bridges, whose modification would likely prevent the conjugation strategy due to their role on protein folding. The authors instead, inserted another residue with rationally depicted positions to allow the best functionalization approach. Inserted cysteine residue at position 88 enabled the RNase A conjugate to remain active and to evade a common proteinaceous inhibitor, which validated its therapeutic potential in a drug delivery system. In another example, the dihydrofolate reductase EcDHFR was found to be stabilized by glycosylation followed by previous insertion of a cysteine residue at position 87 and functionalization by iodoacetamide sugars (Iwakura et al., 1995; Tey et al., 2010). This region revealed interesting biophysical properties that allowed further exploitation. Once more, the protein has two naturally occurring cysteine residues, which upon mutation appeared to not impact the enzymatic activity, that were not exploited by the authors. Since EcDHFR and human DHFR are important targets in infectious diseases and cancer (Raimondi et al., 2019), exploitation of this site might provide an alternative route to design new inhibitors. Other low abundant amino acids such as tryptophan, methionine, tyrosine and histidine are also pursued in genetically engineered systems for which rational positioning within the protein target permit sites for unique chemical handles (Hoyt et al., 2019; Isenegger and Davis, 2019). On the other hand, modification approaches targeting naturally occurring residues offer more advantages to selected bioconjugation strategies since they avoid the genetic engineering required to arrive at suitable bioconjugation conditions (Spicer and Davis, 2014; Koniev and Wagner, 2015; Matos et al., 2018). Taking advantage of naturally occurring residues in a bioconjugation strategy permits the rapid development of a protein-drug conjugate. For instance, the bioconjugation of RNase A, Lysozyme C, and the peptide hormone somatostatin (SST-14) with a biotin tag using *N*- hydroxysuccinimide (NHS) esters (Chen et al., 2012). Site-selectivity was readily achieved at the naturally occurring residues Lys1 in RNase A and Lysozyme C, and at Lys9 in SST-14 which led to stable bioconjugates able to retain activity. It is worth to notice, however, that the bioconjugation at Lys1 in RNase A slightly disrupted the protein activity, which might indicate the functional relevance of this residue. Interestingly, individual reactivity played an important role in this strategy since it was observed that it is required more than solvent accessibility to achieve a modification at a specific residue.

## Site-Specific Protein Modification

Although promising in protein-based technologies, protein modification methods still face many hurdles. This is the result of reactions being more site-selective than site-specific (Krall et al., 2016). A site-specific reaction allows the modification of a specific amino acid over other amino acids with the same side chain (e.g., a single cysteine residue modified over other accessible cysteine residues) (Figure 1). Several methods have been developed to enhance site-specificity. For example, tryptophan residues modified with 9-azabicyclo[3.3.1]nonane-3-one-N-oxyl derivatives. In this case Trp62 of Lysozyme C was specifically modified over five other Trp residues (Trp28, Trp63, Trp109, Trp112, and Trp124). The reaction was not only site-specific as it was site-selective, since cysteine and tyrosine residues remained intact (Seki et al., 2016). Lysozyme C is a common enzyme in biotechnology applications, therefore, exploiting bioconjugation strategies could lead to improved products also in catalysis and biomedical applications (Wei et al., 2011). Trp62 modification is explained by its location since according to the authors it is the least sterically hindered among Trp residues. Functionalization of this site did not cause any changes in structure or activity, highlighting its potential use in bioconjugation. Tyrosine residues modified by luminol derivatives in the presence of H_2_O_2_ and hemin, here the protein model bovine serum albumin was selectively modified on exposed tyrosine residues, and Tyr400 the site of two modifications (Sato et al., 2015). The authors suggested that optimized protocols might lead to site-specific modifications. Histidine residues modified by Pt(II)-driven and Ru(II)-driven complexation (Solomatina et al., 2017) or by “chemical linchpins” in a linchpin-directed modification (LDM) (Adusumalli et al., 2018). In these examples, His68, the only histidine residue in ubiquitin (UBQ) was selectively modified and facilitated the developed site-specific bioconjugations. Nonetheless, in the Pt(II)-driven and Ru(II)-driven complexation the conjugation significantly changed the conformation of UBQ as observed by NMR comparative studies, whereas in the LDM approach it remained unperturbed. The two different methods highlight that the His68 environment is certainly important for the protein structure and might as well be exploited for purposes other than bioconjugation. Since UBQ is relevant in disease settings including Parkinson's disease (Walden and Muqit, 2017), exploiting this site might offer therapeutic advantages. Methods to enhance site-specific lysine modification have also been accomplished (Chilamari et al., 2017). In this work, by using a multi-component reaction containing an aldehyde, an acetylene and Cu-ligand complex a propargylamine handle was efficiently conjugated to nine different proteins at a single lysine residue while their enzymatic activity remained largely unaltered. Comparatively, the aforementioned examples shared the same feature, to produce active protein-drug conjugates. Thus, reports that explain loss-of-function promoted by the modification strategy are uncommon. Furthermore, there are relatively few examples in which protein modification methods are performed in disease-relevant POIs rather than model proteins, and ADCs are certainly the most common.

## Site-Specific Protein Modification in The Development of ADCs

**Figure 2:**
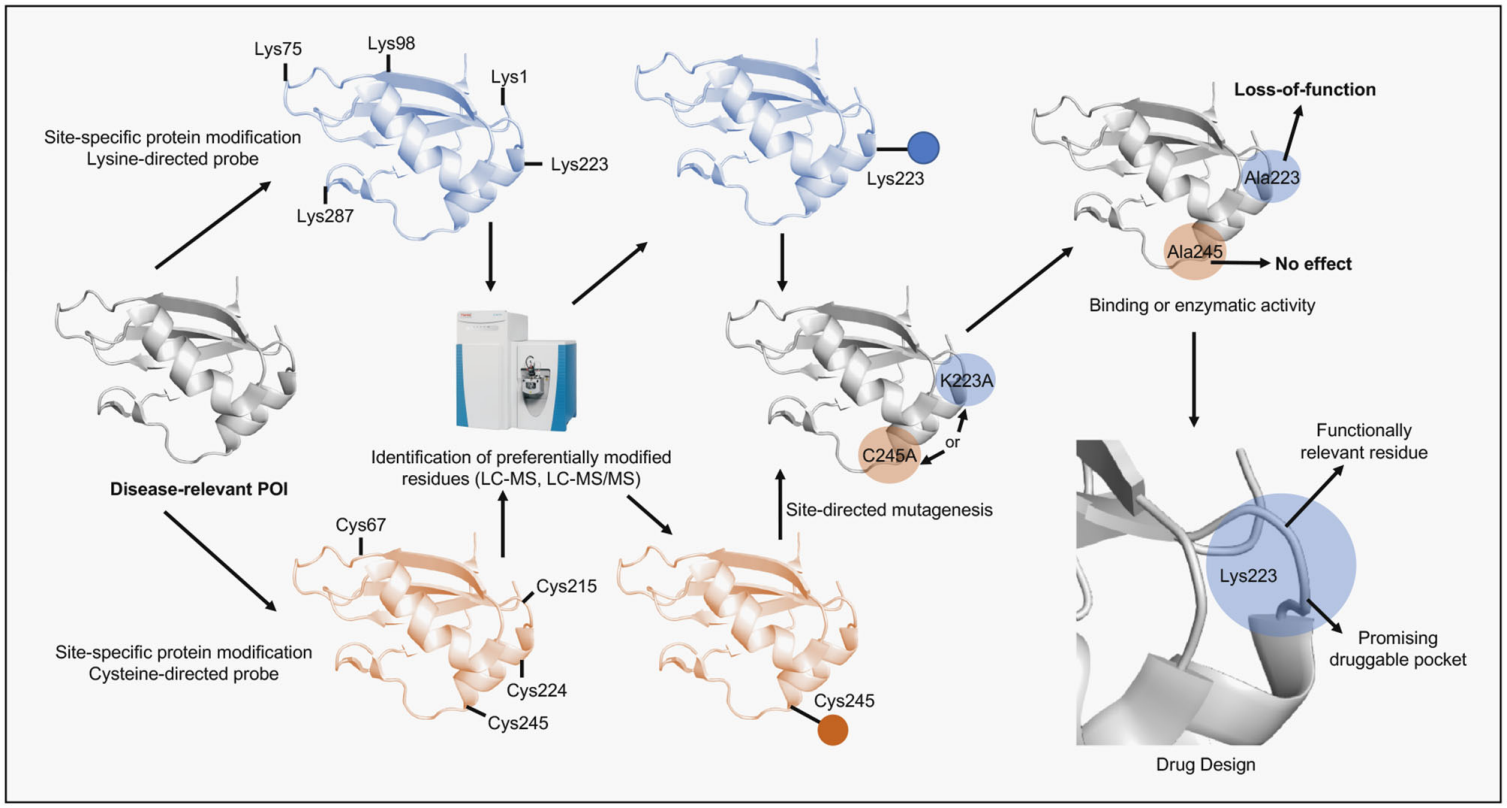
Scheme of the alternative application of site-specific protein modification approaches proposed in this perspective. A disease-relevant protein (POI) is selected, recombinantly expressed and purified. Next, the POI is submitted to reactions containing a site-specific chemical probe (e.g., lysine- or cysteine-directed probes). The reactions are performed in mild conditions to preserve protein folding. Subsequently, the solution is digested by proteases and analyzed by LC-MS/MS in order to identify probe-modified peptides and reveal the site of the specific modification. Once identified the residue is then replaced by alanine using site-directed mutagenesis and the protein mutant, following expression and purification, is assessed according to its function (e.g., binding or enzymatic). If the protein activity is impaired by the mutation then the residue is considered functionally relevant. Lastly, the site surrounding the identified residue can be revealed using structural biology tools, validated as druggable pocket and used in computer-aided drug discovery programs.

ADCs are the main hub gathering most of the site-specific protein modification approaches due to their therapeutic potential in cancer (Chau et al., 2019). ADCs combine the specific recognition of tumor-expressing antigens by antibodies with the cellular toxicity of drugs (also named payloads) into a targeted therapeutic construct. Many aspects of ADCs design are essential for their function and are not covered in this perspective but have been thoroughly addressed in recent reviews (Rodrigues and Bernardes, 2016, 2018; Thomas et al., 2016; Chau et al., 2019). We focused instead, in the linker development, the part of the ADCs that require site-specific protein modification methods. Linkers are especially important and required careful consideration if good stability, pharmacokinetics and pharmacodynamics are envisioned (Jain et al., 2015; Mccombs and Owen, 2015; Tsuchikama and An, 2018). To allow the ADC to achieve maximum therapeutic potential linkers have to connect the payload to the antibody and be able to resist premature cleavage (cleavable and non-cleavable linkers) whilst promoting the rapid release (cleavable linkers) of the payload once the ADC is internalized, a multifaceted task (Beck et al., 2017). Linker chemistries are plenty and focused mostly on modification of lysines and cysteines. Initially, stochastic modification of lysines was employed but proved to be rather suboptimal, as some of the random modifications altered the ADCs' antigen-binding properties, which highlighted the need to control the exact site of modification (Panowski et al., 2014; Matos et al., 2018). In this context, cysteine conjugation offers more space to decrease linker heterogeneity and allow site-specific modifications due to its relative low abundance. Methods to achieve these features include the use of next generation maleimides (NGMs) to produce ADCs via functional disulfide bridging and the development of cysteine-specific carbonylacrylic reagents (Nunes et al., 2015; Bernardim et al., 2016, 2019). Bioconjugation strategies using cysteines have been the choice in many ADCs, FDA-approved or currently under trials (Jain et al., 2015). In general, these enhanced site-specific modification approaches continue to be largely employed in the development of protein-based technologies in which the POI is the product. Here, activity integrity and product stability are the most common aims. It is interesting to notice that in the pursuit of the best chemical probes to achieve site-specific modifications chemical biologists have developed compounds that can be used beyond the protein-conjugates field (Blagg and Workman, 2017). For example, the loss-of-function promoted by some chemical probes as a result of an unintended specific modification can indicate the role of the modified amino acid in the POI's activity or structure while revealing possible “druggable pockets.” This information can then be exploited in drug design approaches directed to POIs involved in disease, including cancer. Moreover, the same chemical probe can yet be optimized to be used as a small drug-like molecule (Figure 2).

## Unveiling Druggable Pockets With Site-Specific Protein Modification

There are few examples in which site-specific modification approaches were used to reveal druggable pockets. In these examples, authors tried to understand the reasons for the preferential targeting of a specific cysteine or lysine residue by a chemical probe over other equally reactive and accessible residues and the consequent loss-of-function induced by the modification. In turn, such analysis revealed interesting new motifs, which prompted interest in drug design platforms. The protein tyrosine phosphatase A from *Mycobacterium tuberculosis* (MPtpA) is one POI where this strategy was employed and revealed the site surrounding Cys53 as a promising druggable pocket. PtpA has three cysteine residues and incubation with a 2,5-dibromohexanediamide (DBHDA) probe led to the Cys53's preferential modification over the catalytic Cys11 and the “backdoor residue” Cys16. Cys53 proved to have a role protecting against overoxidation the catalytic Cys11 because its modification critically impaired PtpA's ability to remain active after exposure to a high oxidative environment. Similar results were also obtained with the protein tyrosine phosphatase YopH from *Yersinia enterecolitica* in which Cys259 was preferentially modified over the catalytic Cys403. These examples highlighted the site-specific modification of non-catalytic cysteines and presented very promising sites for the allosteric inhibition of bacterial phosphatases (Bertoldo et al., 2017, 2018). Since most drug discovery approaches rely on competitive inhibitors targeting the catalytic pocket, these sites might allow an alternative route to develop irreversible and highly selective inhibitors. The site-specific PEGylation of the fibroblast growth factor 2 (FGF2) at Cys27 or Cys129 also led to drastic decrease in the binding to its receptor (Zhao et al., 2020). In this work, FGF2 which has 4 naturally occurring cysteine residues, was mutated in order to remove two surface exposed cysteine residues. Subsequently, engineered cysteine residues were introduced at several positions to test optimal bioconjugation conditions. K27C and R129C mutants displayed a severely disrupted activity upon cysteine-mediated PEGylation, suggesting sites around the receptor binding region (Lys27) and the heparin binding region (Arg129) to be avoided in future bioconjugation strategies. On the other hand, the FGF2/FGF2R axis has been implicated in cancer progression and proposed as a promising target for therapeutics (Akl et al., 2016; Chakraborty et al., 2017; Giulianelli et al., 2019), therefore, these sites could potentially be exploited as druggable pockets. Intriguingly, some site-specific modification approaches can lead to gain-of-function as opposed to loss-of-function. For example, the Hsp70's Cys622 was preferentially modified by a carbonylacrylic probe over other four free cysteines (Cys37, Cys287, Cys326, Cys593) which revealed a highly promising druggable site (Lindstedt et al., 2019). This site was exploited by a conjugation strategy that enabled the modulation of Hsp70 activity. Drugging Cys622 with a *(E)-N*-(2-((7-nitrobenzo[c][1,2,5]oxadiazol-4-yl)amino)ethyl)-4-oxo-4-phenylbut-2-enamide (caaNBF) led to a significant increase in Hsp70 anti-aggregation activity. This residue is located in the substrate binding domain and demonstrated the lowest pK_a_ value, which explained its site-specific modification by the chemical probe. The region surrounding Cys622 presents an interesting pocket for drug discovery approaches aimed at enhancing the Hsp70 activity. Since this conjugate proved to function in an *in vivo* model of Parkinson's disease (PD) it might present a further step into the developing of new therapeutic interventions against PD. Examples involving the site-specific modification of lysine residues also show loss-of-function. In spite the chemo- and regio-selectivity displayed by lysine-directed probes, impaired protein activity and structure still results from the modification. Lys573 in the recombinant human serum albumin (rHSA) is such an example, with 59 lysine residues spread over the protein structure Lys573 was found to be preferentially modified when incubated with a sulfonyl acrylate probe (Matos et al., 2018). HSA is the most abundant plasma protein and its recombinant variant has several applications including drug delivery (Sleep, 2015). Nevertheless, modification at this site disrupted rHSA binding to FcRn receptor. This is another example which corroborates the idea that chemical probes react preferentially with low pK_a_ residues and that often these residues are functionally relevant, which is the case of Lys573 (Sand et al., 2015). Despite the fact that disrupting HSA binding to FcRn has no clear therapeutic applications, the concept is still valid. Using site-specific chemical probes in proteins which modulation (either loss- or gain-of-function) produces a clinically relevant response might allow the discovery of hitherto unknown druggable pockets. Furthermore, exploitation of such pockets can yet produce innovative and specific drugs which target relevant residues whilst sparing other highly nucleophilic ones. Since there is a resurgence in the development of covalent inhibitors, applying this method to aid drug discovery programs seems highly appropriate.

## Concluding Remarks

It is our understanding that site-specific modification approaches might offer an alternative avenue in the forefront of covalent inhibitor design. Covalent inhibitors have reappeared as valid choices in cancer treatment recently with examples such as ibrutinib, that targets site-specifically a cysteine residue near the catalytic pocket of the Bruton's tyrosine kinase (Tucker and Rule, 2015). Although, careful consideration has to be taken when selecting a chemical probe (Blagg and Workman, 2017), it is possible to take advantage of the information they provide as a mean to pursuit innovative therapies. The examples described in this perspective demonstrate that site-specific chemical probes can be used to reveal functionally relevant residues, which can be located in a hitherto unknown druggable pocket. The unique features of the newly discovered pocket combined with the functional relevance of the amino acid might afford an elegant platform to develop highly selective anti-cancer drugs. In addition, the probes can be repurposed to function as inhibitors by fully exploiting these features.

## Data Availability Statement

The original contributions presented in the study are included in the article/supplementary material, further inquiries can be directed to the corresponding author/s.

## Author Contributions

JB conceived and wrote the article with contributions from DM. SH edited the manuscript. All authors reviewed and approved the submitted version.

## Conflict of Interest

The authors declare that the research was conducted in the absence of any commercial or financial relationships that could be construed as a potential conflict of interest.
